# New Topical Therapy for Provoked Vestibulodynia: Improvement of Psychological and Sexual Well-Being

**DOI:** 10.3390/ijerph20031931

**Published:** 2023-01-20

**Authors:** Francesco De Seta, Patrizia Ianniello, Stefania Carlucci, Luigi Nappi, Felice Sorrentino, Guglielmo Stabile

**Affiliations:** 1 Institute for Maternal and Child Health, IRCCS Burlo Garofolo, 34100 Trieste, Italy; 2 Department of Medical, Surgical and Health Sciences, University of Trieste, 34100 Trieste, Italy; 3 Department of Medical and Surgical Sciences, Institute of Obstetrics and Gynecology, University of Foggia, 71121 Foggia, Italy

**Keywords:** vulvodynia, provoked vestibulodynia, new therapy, sexual well-being, topical therapy, vulvar vestibulitis syndrome

## Abstract

Vulvodynia is a vulvar discomfort that occurs in the absence of any specific, clinically identifiable disorder. Few therapies have shown to be effective for the treatment of vulvodynia. In our recently published study, we tested a drug-free gel in women affected by vulvar vestibulitis. It is a cosmetic gel which acts locally without any metabolic, pharmacological or immunological effect. In order to further promote the validity of this new product, in this manuscript we analyzed the results obtained from the administration of four questionnaires in the same two groups of women affected by PVD and treated with a placebo and the new product. The questionnaires used: Female Sexual Function Index Scoring (FSFI), Female Sexual Distress Scale (FSDS), Hospital Anxiety and Depression Scale (HADS), and health-related quality of life measured by SF-36 (SF-36). The results obtained by this current analysis showed that the new gel has also proven benefits on women’s quality of life and sexual function, including improvements in arousal, desire, orgasm and satisfaction.

## 1. Introduction

Vulvodynia is a vulvar discomfort that occurs in the absence of any specific, clinically identifiable disorder.

In 2015, representatives from the International Society for the Study of Vulvovaginal Disease, the International Society for the Study of Women’s Sexual Health, and the International Pelvic Pain Society developed a consensus terminology and classified persistent vulvar pain into two categories: vulvar pain caused by a specific disorder and vulvodynia [1].

Vulvar pain caused by a specific disorder can be due to infectious, inflammatory, neoplastic, or neurologic processes, and the treatment of these causes solves the vulvar pain. By contrast, vulvodynia is defined as vulvar pain of at least three months duration that has no identifiable cause; the lack of a specific cause makes more difficult its own treatment [2].

According to the latest consensus, vulvodynia can also be classified considering the localization (localized, generalized or mixed), the onset (primary or secondary), the temporal pattern (intermittent, persistent, constant, immediate) and the provocation (provoked, spontaneous, mixed) [3].

Provoked vestibulodynia (PVD) is the prevalent subtype of vulvodynia, and 12% of women are afflicted by this pathology at least once in their life [4].

PVD involves pain that is localized to the vulvar vestibule and is triggered by contact to the affected area. The pain can become particularly intense during vaginal penetration. Indeed, dyspareunia is the main disorder of women with PVD, and PVD is considered to be the most common type of sexual pain condition among women of reproductive age. PVD has a negative impact on women’s sexual activity. Women with PVD complain that sexual intercourse is always painful and often impossible and this leads to a reduction in sexual desire and arousal, making orgasm difficult [4]. Controlled studies show that affected women report more sexual distress, poorer sexual function and feelings of inadequacy as a sexual partner. Anxiety and depression are typical consequences of vulvodynia, alongside the fact that depression may interfere with women’s sexual function.

Partners are also negatively affected by PVD, with poorer erectile function and sexual satisfaction [5].

Vulvodynia is studied through validated questionnaires that may help healthcare professionals in interpreting and scoring the type of pain reported by the patient.

Few therapies have shown to be effective for the treatment of vulvodynia. In a recently published study, we tested a sexual hormone-free gel in women affected by vulvar vestibulitis [6]. It is a cosmetic gel which acts locally without any metabolic or immunological effect.

Our data suggest that the new product may be effective in promoting symptom remission in vulvitis; it promotes the natural healing of vulvar skin and vestibular mucosa by enhancing both the hydration and an adequate lubrication of these tissues. Furthermore, zanthalene (antipruritic and anti-inflammatory), glycyrrhetinic acid(anti-inflammatory) and Opuntia ficus-indica (soothing and anti-inflammatory) mixed with Calendula officinalis quickly reduce inflammation and itching, bringing quick relief (these are preliminary data deriving from a 3 month follow-up and will be validated in the future). In order to further promote the validity of this brand new product, in this manuscript we analyzed the results obtained by the administration of four questionnaires in the same two groups of women affected by PVD and treated with either a placebo or the new product. The questionnaires were Female Sexual Function Index Scoring (FSFI), Female Sexual Distress Scale (FSDS), Hospital Anxiety and Depression Scale (HADS), and health-related quality of life measured by SF-36 (SF-36), and were administered to fertile women diagnosed with vulvar vestibulitis and treated with the active compound.

## 2. Materials and Methods

Our study is a double-blind, placebo-controlled, monocentric, prospective, randomized study, which was conducted in Bangalore, Karnataka, between September 2021 and October 2021.

This trial was registered prospectively at the Clinical Trials registry, India, with number CTRI/2021/07/034644.

The study was conducted in accordance with the principles enunciated in the Declaration of Helsinki 2013 and the Central Drugs Standard Control Organization (CDSCO) under the New Clinical Trial Rules and Regulations 2019, Ethical Guidelines for Biomedical Research on Human Participants 2006 of the Indian Council of Medical Research (ICMR).

Our primary endpoint was to demonstrate that the new compound is superior to a placebo and is efficacious in the improvement of women’s sexual functioning and in the reduction of sexual pain.

We tried to establish the effectiveness of the product in the sexual sphere by subjecting the patients to different questionnaires:

1. The Female Sexual Function Index (FSFI) is recognized as an excellent tool for assessing female sexuality and screening female sexual dysfunction [7].
2. The Female Sexual Distress Scale (FSDS), a valid measure for assessing sexually related distress in women [8].
3. The quality of life (QoL) questionnaire (SF-36) is an oft-used, self-reported measure of health. It comprises 36 questions which cover eight points of health [9].
4. The Hospital Anxiety and Depression Scale (HADS) is a self-report questionnaire designed to screen anxious and depressive states in patients in non-psychiatric settings [10].

We enrolled a total population of 40 subjects in the reproductive stage (age range: 18–45 years) diagnosed with PVD. Women were recruited into the study after signing an informed consent form. To guarantee the blindness of the study, the placebo group patients obtained a product with the same characteristics of the active product and contained in an identical box.

Inclusion criteria were:

1. Patient able to give informed consent; 2. Age between 18 and 45 years; 3. Patients diagnosed with PVD who had at least one of the following: a history of vulvar pain/vulvar vestibule erythema/vulvar tenderness upon the cotton swab test (“Friedrich’s Criteria”)/presence of symptoms for ≥3 months and no more than 1 year; 4. Patients with coexisting vulvovaginal candidiasis, on maintenance with azole and negative follow-up fungal cultures; 5. Patients not taking other therapy for PVD for at least a month before enrolment; 6. Acceptance of the obligation to return to all planned visits and follow-ups.

Exclusion criteria were: 1. Pregnant and lactating women; 2. Subjects with a clinically significant history of comorbid conditions (diabetes, immunodeficiency, HIV, HPV), neuropathology, psychological disorders, other pathologies that could cause chronic pain, atrophic vaginitis, dermatitis such as vulvar dystrophy, eczema or pathogens such as culture proven *Candida* spp. or Herpes simplex; 3. Subjects on hormone replacement therapy or chemotherapy or radiotherapy; 4. Patients who were participating in other clinical trials or who had participated in clinical trials within the past 3 months; 5. Genital bleeding of unknown cause; 6. Known history of malignancy; 7. Known history of drug or alcohol abuse; 8. Psychiatric disorders precluding informed consent; 9. Any other medical condition that the investigators felt would compromise the study; 10. Known hypersensitivity to, or intolerance of, the study products or their formulation excipients.

Both study products, the new compound and the placebo, were coded with a computer-generated simple randomization schedule. The recruited patients were randomly divided into 2 study samples: Group 1 received the active product, and Group 2 received the placebo product.

The placebo product was made with the same pH and the same excipients but without active excipients: Propulsave, zanthalene, hyaluronic acid, glycyrrhetinic acid, Calendula officinalis and Opuntia ficus-indica.

We estimated that a sample of 18 patients per group achieved a power greater than 80% to reject the null hypothesis. The level of significance was set at 5% (two-sided). In conclusion, allowing for a 10% dropout, the total sample size was 40.

An accurate medical history was recorded. We performed laboratory tests with a urine pregnancy test during the screening visit to rule out any hidden conditions.

For each patient, the following steps were carried out:

- At day 0, a baseline visit was conducted (data have already been presented in our previous work). Subjects taking both the active compound and the placebo were asked to apply the emulgel twice a day (finger dose), in the morning and at bedtime, to the vulva and vestibule for 2 consecutive weeks. For the following 2 weeks, we requested them to administer the products only in the evening for 3 times a week.
- Questionnaire for FSFI, FSDS, quality of life questionnaire with SF36, and the HADS questionnaire were completed and recorded at baseline visit and at visit 5, 4 weeks after recruitment and initial treatment.

All visits were performed and data collected by the two same investigators. This study was double-blind; for this reason investigators did not know the type of product used by the patients.

The Wilcoxon signed rank sum test was used for statistical analysis of continuous data (*p*-value).

## 3. Results

We enrolled forty subjects in our study. In Group 1 (active), patients enrolled were aged between 19 and 45 years, and in Group 2 (placebo), patients enrolled were aged between 30 and 45 years. The demographic characteristics are shown in Table 1.

Considering data from FSFI, FSDS, HADS and HQRL SF-36, we matched data obtained from the two groups during the initial screening and the visit after 4 weeks from the beginning of the treatment.

**Figure 1:**
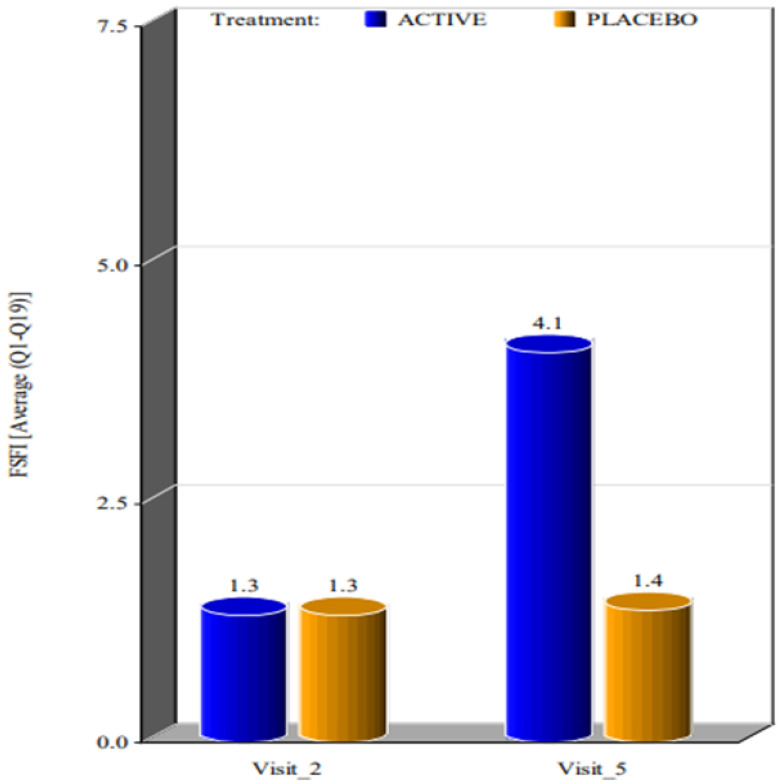
Mean value of FSFI considering the 19 questions at the first visit and at visit 4 weeks after the beginning of the treatment. (Visit 2 corresponds to the visit performed on day 0, as Visit 1 corresponds to the day of laboratory tests).

The two groups reported significant differences in the improvement of FSFI parameters. By week 4 the subjects in the active group showed improvement in all FSFI parameters when compared to the placebo group, which showed a minimal improvement from the baseline (Figure 1).

In screening, minimum FSFI (full scale score range) scoring was 8 and maximum scoring was 11 for subjects in both the active group and the placebo group. In week 4, the minimum scoring was 24 and maximum scoring was 35 for subjects in the active group, while in subjects in the placebo group it was 8 and 12, respectively. The *p*-value for arousal, desire, full scale score range, lubrication, orgasm, pain, satisfaction and for average (Q1–Q19), which was estimated by the Wilcoxon signed rank sum test, showed that there was a very high statistical significance from baseline to week 4 (*p* value < 0.0001 for all parameters in FSFI) between active and placebo groups.

**Figure 2:**
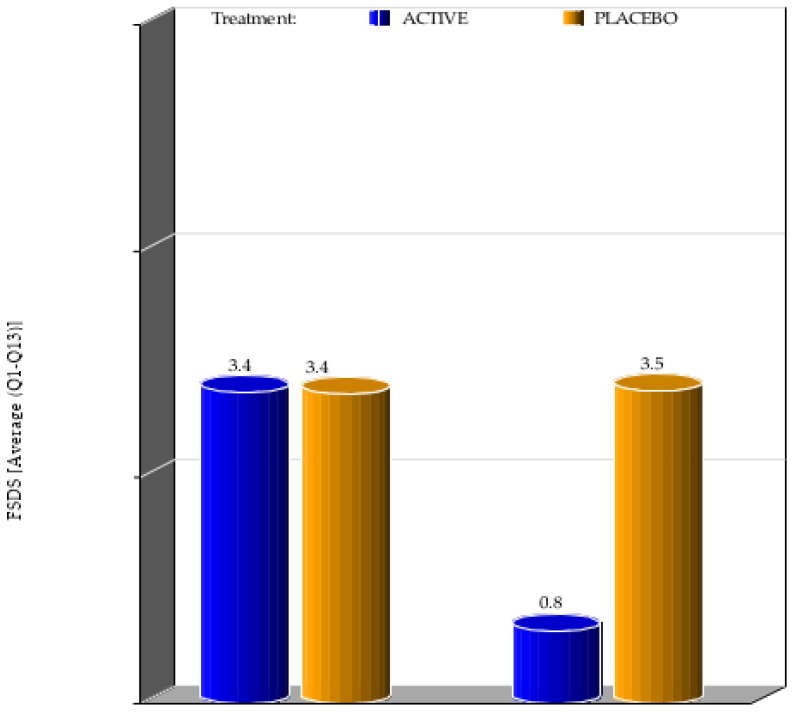
Mean value of FSDS considering the 13 questions at the first visit and at visit 4 weeks after the beginning of the treatment.

A significant difference was registered between the two groups in the improvement of FSDS parameters. While in the placebo group, there were no significant differences in the FSDS score between the screening visit screening and the visit after 4 weeks of treatment, in the active group there was a significant improvement with an average value which passes from 3.4 at the first visit to a value of 0.8 at the final visit (Figure 2). In week 4, the minimum scoring was 0 and maximum scoring was 1 for subjects in the active group, for subjects in the placebo group it was 3 and 4 respectively. This showed that the product worked very well in making a positive change for FSDS (average Question 1–13) when compared to the placebo. The *p*-value for FSDS which was estimated by the Wilcoxon Signed rank sum test, showed that there was a very high statistical significance from baseline to week 4 (*p* value < 0.0001) between active and placebo groups.

**Figure 3:**
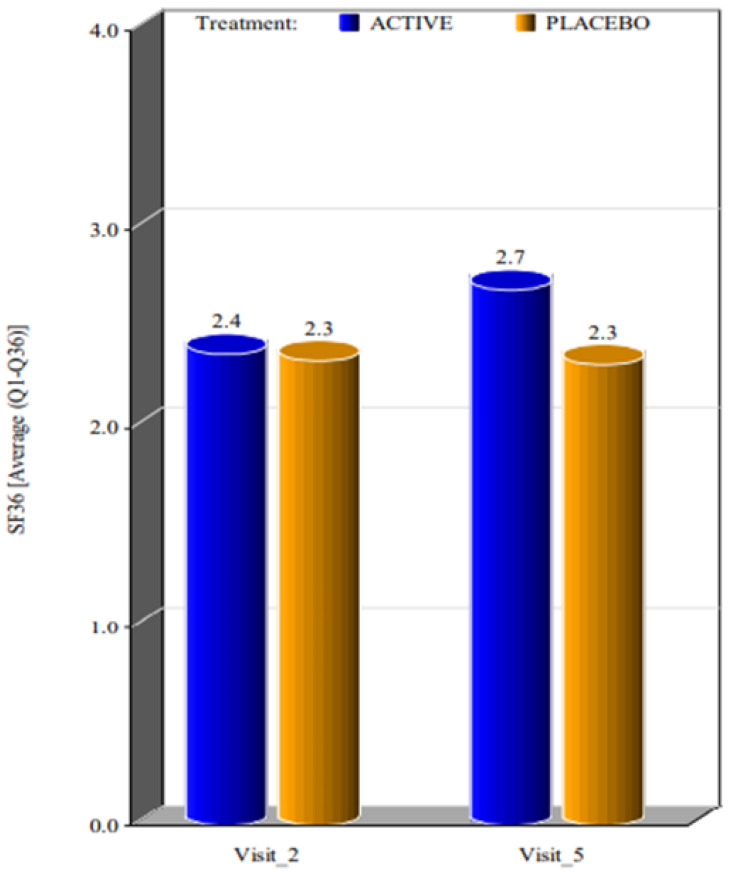
Mean value of SF36 considering the 36 questions at the first visit and at visit 4 weeks after the beginning of the treatment. (Visit 2 corresponds to the visit performed on day 0, as Visit 1 corresponds to the day of laboratory tests).

Considering the questionnaire HRQL SF-36 (Questions 1–36), at the baseline visit the scoring was the same for both groups. After 4 weeks of treatment, the subjects in the active group showed very good improvement in HRQL SF-36 (Average Q1–Q36) when compared to the placebo group, which did not show any improvement from the baseline (Figure 3). This showed that the product worked very well in effecting a positive change for HRQL SF-36 when compared to the placebo. Certainly, our results are partial and take into consideration a limited follow-up time, but the results seem to be encouraging. It is important to keep in mind the negative result of the control sample, on which treatment failure probably had a negative psychological impact. This effect requires caution in defining a treatment with a short follow-up as curative.

**Figure 4:**
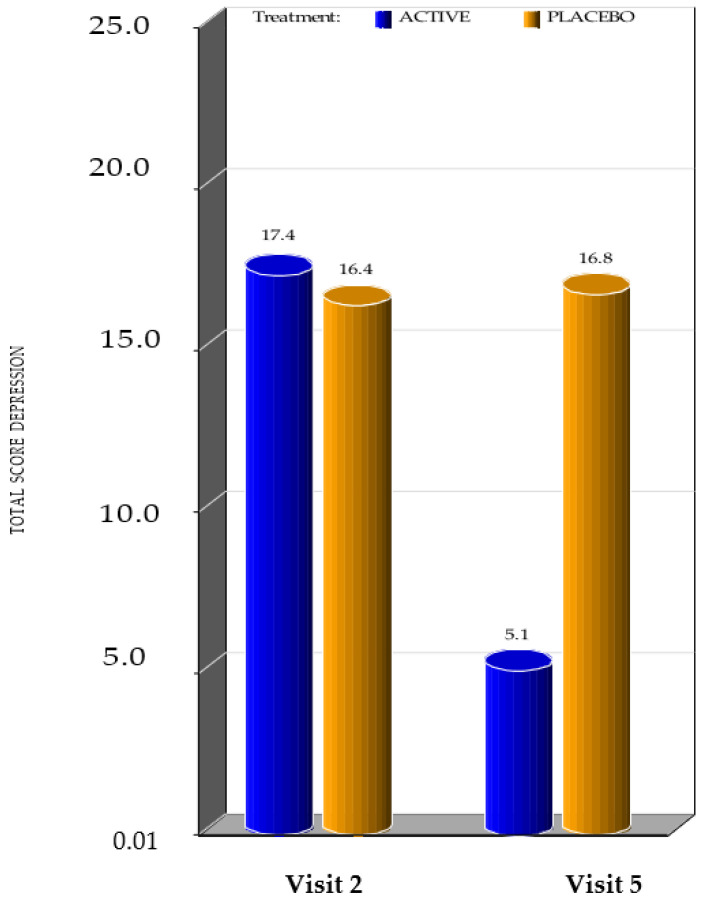
Mean value of ADHS at the first visit and at the visit 4 weeks after the beginning of the treatment. (Visit 2 corresponds to the visit performed on day 0, as Visit 1 corresponds to the day of laboratory tests).

At the baseline visit, both active and placebo group subjects had an ADHS (Total score anxiety and Total score depression) with a high score. The scoring was the same for both groups. By week 4, the subjects in the active group showed very good improvement in ADHS (Total score anxiety and depression) when compared to the placebo group, which did not show any improvement from the baseline. (Figure 4)

In screening, the minimum ADHS (Total score depression) score was 12 and the maximum score was 21 for subjects in both the active group and placebo group. In week 4, the minimum scoring was 2 and the maximum scoring was 8 for subjects in the active group, while in subjects in the placebo group it was 12 and 21, respectively.

The *p*-value ADHS (Total score for depression anxiety) which was estimated by the Wilcoxon signed rank sum test, showed that there was a very high statistical significance from baseline to week 4 (*p* value < 0.0001 and <0.0001 respectively) between active and placebo groups.

This showed that the new gel worked very well in improving ADHS (Total score depression and anxiety) when compared to placebo.

## 4. Discussion

PVD is often associated with comorbid physical and psychological conditions, including depressive and anxiety disorders [11,12,13,14].

Women with PVD suffer from sexual dysfunction and associated distress, including difficulties with desire, arousal and orgasm [15,16]. This condition unfortunately creates psychological problems even in the partners of affected patients, worsening the social weight of this pathology. The worsening of the couple’s relationship over time, together with the disappointment related to the ineffectiveness of the therapy, could explain the worsening of the score in the various questionnaires carried out 4 weeks after the start of therapy.

In the literature, different therapies for vulvar pain treatment have been compared, such as topical lidocaine, corticosteroids, or capsaicin for the treatment of localized vestibular pain; but also low oxalate diet, tricyclic antidepressants (TCAs), selective serotonin re-uptake inhibitors (SSRIs), systemic and topic hormones and vestibulectomy. Nonetheless, there is insufficient evidence defining the effectiveness of these therapies. Moreover, it should be also considered that these drugs can lead to adverse effects, especially if systemically administered. A valid alternative route of drug delivery could be the vaginal administration, which provides targeted therapy with decreased systemic adverse effects, reducing patients’ fears and increasing compliance with therapy [15,17].

In our recent study, we have demonstrated how the new compound, a vaginal drug- and sexual hormone-free gel, when used in women with vulvitis, affects the natural healing of vulvar skin and vestibular mucosa, acting on the lubrication and rehydration of these tissues. This new formulation was shown to have statistically significant effects (*p* value ≤ 0.05) in reducing all symptoms of Friedrich’s criteria and Marinoff dyspareunia and displayed a good tolerability and safety.

Therefore, this new product should be considered as a new possible option in promoting symptom remission of vulvitis [6].

With these results we have demonstrated that this therapy leads to an improvement in psychological and sexual well-being in the patients treated. This is shown by the improvement in the score in all the questionnaires to which they were submitted (FSFI, FSDS, HADS and HQRL SF-36). On the contrary, in the group of patients treated with the placebo, we can observe either the same score in the second visit or a slight deterioration, possibly due to a to a worsening of symptoms (as shown in our previous study) [6] or a lack of improvement in symptoms that persist over time leading to a worsening of the psychological state of the patients. The disappointment following therapeutic failure should also be considered in patients who in many cases have already been subjected to various unsuccessful therapies.

The strength of our study is the homogeneity of symptoms at the first visit and clinical history of the two groups. Furthermore, it was a randomized, double-blind and placebo-controlled study. The questionnaires were administered simultaneously for the two groups and by the same two investigators. The questionnaires were previously well explained and described to investigators and patients.

The limitations of our study are represented by the small sample size of the two groups and the difference in age between the two groups. This difference may have affected hormone levels and sexual behavior and desire.

## 5. Conclusions

The new interesting results obtained by this current analysis show that the active compound has proven benefits on women’s quality of life and sexual function, including improvements in arousal, desire, orgasm and satisfaction. Furthermore, this shows that adequate therapy, free from side effects, of vulvodynia leads to the psychological well-being of affected patients, improving their relationship life. On the contrary, a failed therapy leaves the patient in critical psychological condition, in some cases making them worse. However, further and more extensive data on a greater number of patients are needed in order to confirm our results.

## Figures and Tables

**Table 1:** Demographics characteristics of the two group.

Parameter/Statistics.	Group 1 (Active)	Group 2 (Placebo)
**Age (Years)**		
n	20	20
Mean (SD)	31.1 (8.96)	38.4 (5.22)
Median	29.0	38.0
Min, Max	19, 45	30, 45
**Height (cm)**		
n	20	20
Mean (SD)	163.3 (4.57)	165.5 (5.84)
Median	162.0	165.0
Min, Max	157, 172	157, 175
**Weight (kg)**		
n	20	20
Mean (SD)	70.4 (5.45)	70.3 (7.34)
Median	70.0	68.5
Min, Max	61, 84	58, 81
**BMI (kg/m^2^)**		
n	20	20
Mean (SD)	26.427 (2.3543)	25.693 (2.8493)
Median	27.195	26.220
Min, Max	22.65, 30.84	21.16, 31.64
**Past Medical History, n [%]**		
Nil	20 (100.0)	20 (100.0)
Type 2 Diabetes	0 (0.0)	0 (0.0)
**Treatment History, n [%]**		
Nil	20 (100.0)	20 (100.0)
**Co-morbid Conditions, n [%]**		
Nil	20 (100.0)	20 (100.0)
**Concomitant Medications, n [%]**		
Nil	20 (100.0)	20 (100.0)

Legend: BMI (body mass index), Min (minimum), Max (maximum).

## Data Availability

The original contributions presented in the study are included in the article; further inquiries can be directed to the corresponding author.
